# Exosomes and Their Role in Cancer Progression

**DOI:** 10.3389/fonc.2021.639159

**Published:** 2021-03-22

**Authors:** Yang Liu, Ke Shi, Yong Chen, Xianrui Wu, Zheng Chen, Ke Cao, Yongguang Tao, Xiang Chen, Junlin Liao, Jianda Zhou

**Affiliations:** ^1^ Departments of Plastic and Reconstructive Surgery, The Third Xiangya Hospital, Central South University, Changsha, China; ^2^ Department of Dermatology, The First Hospital of Changsha, Changsha, China; ^3^ Department of Oncology, The Third Xiangya Hospital, Central South University, Changsha, China; ^4^ Key Laboratory of Carcinogenesis, Ministry of Education, Cancer Research Institute, School of Basic Medicine, Central South University, Changsha, China; ^5^ Department of Dermatology of Xiangya Hospital, Central South University, Changsha, China; ^6^ Departments of Medical Cosmetology, The First Affiliated Hospital, University of South China, Hengyang, China

**Keywords:** exosomes, tumor microenvironment, angiogenesis, EMT - epithelial to mesenchymal transformation, immune regulation, cancer treatment

## Abstract

Exosomes from extracellular vesicles can activate or inhibit various signaling pathways by transporting proteins, lipids, nucleic acids and other substances to recipient cells. In addition, exosomes are considered to be involved in the development and progression of tumors from different tissue sources in numerous ways, including remodeling of the tumor microenvironment, promoting angiogenesis, metastasis, and invasion, and regulating the immune escape of tumor cells. However, the precise molecular mechanisms by which exosomes participate in these different processes remains unclear. In this review, we describe the research progress of tumor cell-derived exosomes in cancer progression. We also discuss the prospects of the application of exosomes combined with nanoengineered chemotherapeutic drugs in the treatment of cancer.

## Introduction

Extracellular vesicles (EVs) are membrane-bound, nanosized vesicles that are released from different cell-types and are able to transport nucleic acids, proteins, and other cellular cargo (1). EVs comprise three main subtypes, including exosomes, apoptotic bodies, and microvesicles, which are differentiated based upon size, release pathway, biogenesis, and function (1–3). The term “exosome” was originally used to describe a vesicle of unknown origin released by cultured cells (4). Later, exosomes were considered to be membrane-bound vesicles released by reticulocytes during differentiation (5). Exosomes range in diameter from 40 to 150nm (6) and have been found to be secreted by many different types of cells (7). Microvesicles, previously known as ‘platelet dust’, were originally described as subcellular material derived from platelets in normal plasma and serum (8). They are produced through the outward budding and fission of the plasma membrane and by releasing some vesicles into the extracellular space (9). The diameter of microvesicles ranges from 50nm to 1 μm, with a maximum of 10μm (3). Microvesicles have been reported to play an important role in cancer progression by mediating intercellular communication (10).

Intercellular communication is extremely important for various cells to adapt to changes in the different intracellular and extracellular environments and can occur at different processes and stages, such as during embryonic development, in response to trauma, and in the maintenance of homeostasis in the organism (11). The mechanism of communication varies between different cells, ranging from direct and close cell-to-cell contact to long-range effects. Biological signals are transmitted through the circulation of bodily fluids, cell membrane particles, and exosomes, and the latter two are generally considered to be a specific and widespread mechanism of transport (12).

During the maturation of multivesicular endosomes (MVES), exosomes are considered to be intraluminal vesicles (ILVs), which are formed via inward budding of the endosome membrane. They are intermediates in the endosome system and are secreted when MVES fuse with the cell surface (13, 14). Exosomes transport receptors, transcription factors, enzymes, extracellular matrix proteins, DNA, RNA, and lipids, to different places to perform different functions. Among the different types of exosomes, tumor cell-derived exosomes play an essential role in the invasion and metastasis of cancer cells (15). Tumor cell-derived exosomes can transmit tumor metastasis signals, determine the direction of cancer cell metastasis, and promote epithelial-mesenchymal transformation (EMT) and angiogenesis. Some exosomes also have immunomodulatory functions and cancer treatment potential. This article will systematically describe the role of exosomes from different sources in cancer progression.

## Role of Tumor Cell-Derived Exosomes in Remodeling the Tumor Microenvironment

Studies on exosomes and their biological functions have improved our understanding of the intercellular communication of exosomes in different cell types. These nanoscale vesicles are effective carriers of the regulatory information of biological macromolecules, and can be further induced and regulated by the receptor cells. The main cell types including fibroblasts, endothelial cells, and immune cells that interact with cancer cells through exosome signaling in the tumor microenvironment. The outcome of the interactions mentioned above relies on the origin of the exosomes and their exosomal cargo (16, 17). Hypoxia-induced acidosis, starvation, and other stress states of the body increase the release of exosomes from tumor cells, leading to changes in the tumor microenvironment, thus promoting the occurrence and development of tumors (18–20).

Proliferation of tumor cells is an essential process for cancer progression, and this process is dependent upon growth factors. Growth factors can also support the tumor microenvironment (21). By releasing exosomes, cells can transmit information to the tumor microenvironment and improve the ability of tumor cells to proliferate.

In addition, tumor cell-derived exosomes can modify the migratory status of recipient malignant cells (22). These exosomes can also regulate the tumor microenvironment by disrupting cell adhesion and stimulating extracellular receptor signaling pathways (23, 24). For example, the cancer-derived adaption of endothelial cells via miR-105 occurs during early premetastatic niche formation. In the case of breast tumor cells, exosome-mediated miR-105 is secreted from metastatic breast cancer cells and, by targeting the tight ligand ZO-1, it breaks down the barrier function of the endothelial monolayer and induces tumor cells to metastasize to distant organs (25). The enhancement of vascular permeability can promote the distant spread and growth of cancer cells. Additionally, exosome cargo can leak into secondary organs and alter cellular physiology toward a prometastatic or tumor-supportive phenotype (25).

Exosomes are considered to be involved in the progression of various precancerous liver diseases, including viral hepatitis, alcoholic liver disease, and even the progression of liver fibrosis, which eventually develops into hepatocellular carcinoma (HCC) (26). For example, after human beings are infected with the hepatitis virus, exosomes containing viral nucleic acids and proteins are released by infected hepatocytes, allowing the virus to be transfected into healthy hepatocytes and leading to the spread of infection. Therefore, exosomes mediate transmission of the hepatitis virus (27).

**Figure 1:**
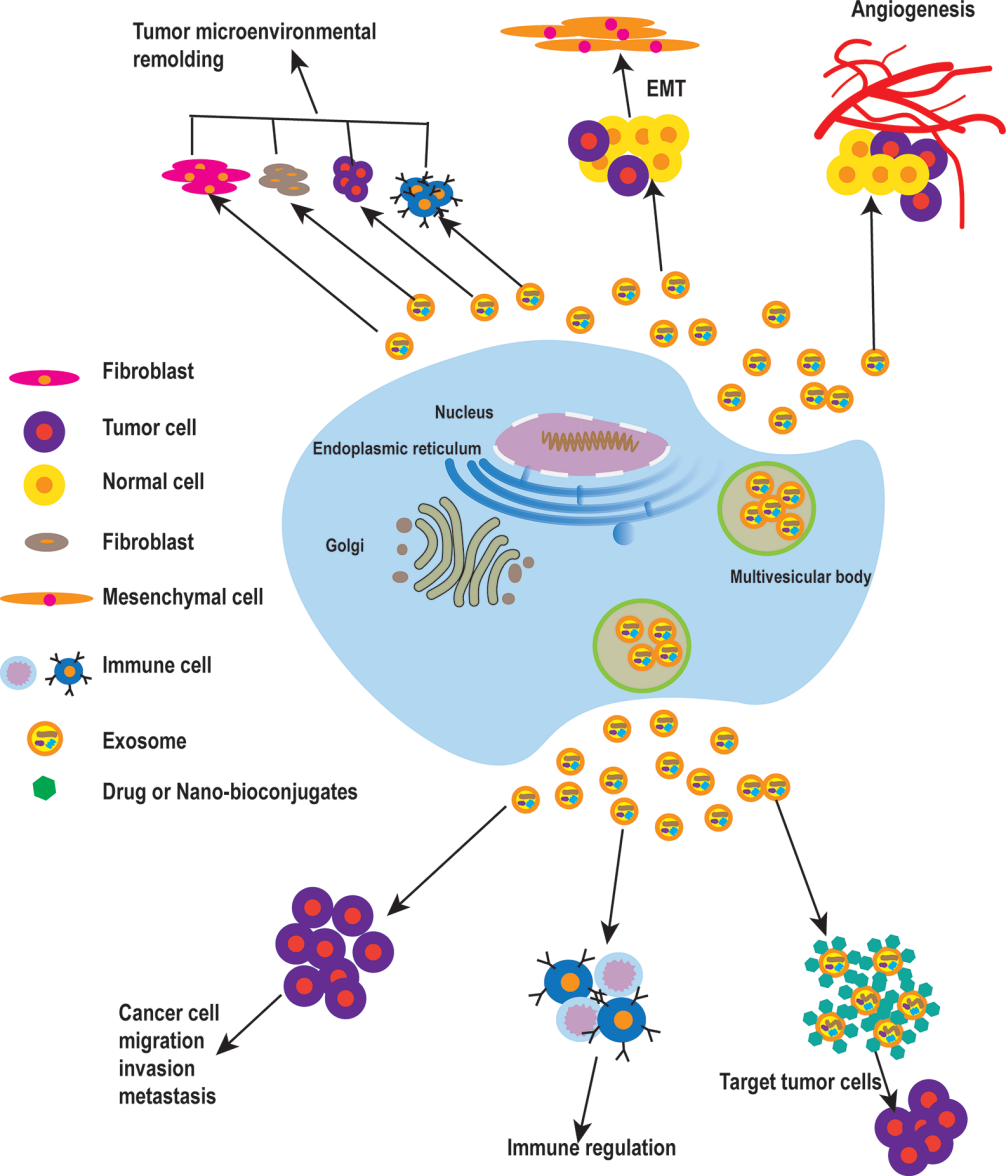
The roles of exosomes in cancer. Tumor cell-derived exosomes play a vital role in the remodeling of the tumor microenvironment, and can promote EMT and increase the motility and invasiveness of tumor cells, leading to tumor migration and metastasis. When tumor cells reach new metastasis sites *in vivo*, tumor cell-derived exosomes can promote the formation of new blood vessels. To enable tumor metastasis, exosomes also can promote tumor metastasis by involving in immune regulation. Finally, exosomes can be used as a carrier for drug delivery in cancer treatment.

Exosomes derived from cancer-associated fibroblasts (CAFs) have been shown to provide certain nutrients to pancreatic and prostate cancer cells and to drive them to glycolysis. Hongyun Zhao et al. demonstrated that CAFs-derived exosomes (CDEs) from patients reprogram cancer cell metabolism by disabling mitochondrial oxidative metabolism and providing *de novo* “off the shelf” metabolites through exosomal cargo (28). Xiaofeng Wang et al. found that co-culturing macrophages with pancreatic cancer cells treated with miR-301A-3p or hypoxic exosomes enhanced the metastatic ability of pancreatic carcinoma cells. These data suggest that pancreatic cancer cells produce exosomes rich in miR-301A-3p in an anoxic microenvironment, which polarizes macrophages and promotes malignant behavior of pancreatic cancer cells (29). Examination of exosomes derived from metastasis-initiating cells (MICs) revealed that these exosomes have the ability to reprogram bystander DC-1 cells by increasing the migration and invasion of DC-1 cells and upregulating MIC-specific genes. This observation indicates that the reprogramming of dormant prostate DC-1 cells may be mediated by MICs-derived exosomes (30). On the one hand, tumor microenvironment-derived exosomes can increase the ability to uptake glucose and enter the TCA cycle and, on the other hand, they can decrease the process of mitochondrial oxidative phosphorylation. These findings may help explain the continued growth of cancer cells in the face of certain hypoxic conditions or reduced sources of nutrients (28). Additionally, these reports indicate that tumor cell-derived exosomes play an important role in the remodeling of the tumor microenvironment (**Figure 1**).

## Tumor-Derived Exosomes Can Promote Epithelial-Mesenchymal Transformation (EMT)

Tumor metastasis is regulated by many factors, including epithelial-mesenchymal transformation, in which the epithelial cells themselves undergo morphological changes that transform them into motor-activated mesenchymal cells. *In situ* tumor cells can also undergo this change, which gives them the ability to invade and metastasize (31, 32).

Qu Z et al. found that hepatocyte-derived exosomes can promote invasion and metastasis of recipient cells, induce decreased E-cadherin expression and increased vimentin expression, and contribute to EMT development. Following treatment of hepatoma receptor cells with MHCC97H and MHCC97L cells-derived exosomes, these hepatoma cells induce EMT through the TGF-β/Smad signaling pathway (33). In metastatic bladder cancer, EMT is associated with an increase in expression of both exosome-derived casein kinase IIα and annexin A2 (34). Huang L et al. found that drug-resistant endothelial cells promote nasopharyngeal carcinogenesis, EMT, and drug resistance through exosomes (35). Exosomes from CAFs can promote EMT in lung cancer cells, and the expression level of SNA1 from exosomes is closely related to EMT in lung cancer cells. CAFs-derived exosomes can promote metastasis and drug resistance of colorectal cancer cells by enhancing cell stemness and EMT (36, 37).Exosome-derived miR-499a-5p promotes cell proliferation, EMT, and migration in lung adenocarcinoma via the m-TOR signaling pathway (38). CD103-positive tumor stem cell-derived exosomes can promote EMT in clear cell renal cell carcinoma where, following miR-19b-3p transfection of tumor cells, tumor stem cells lead to EMT by inducing expression of *PTEN*. In addition, CD103-positive clear cell renal cell carcinoma in serum samples was associated with the development of lung metastases (39). In summary, tumor cell-derived exosomes can promote EMT and increase the motility and invasiveness of tumor cells, leading to the distant metastasis of tumors (**Figure 1** and **Table 1**).

**Table 1:** Roles of exosomes in cancer progression.

Source of exosomes	Molecular	Type	Step of tumorigenesis	Action type	References
Metastatic breast cancer cells	miR-105	miRNA	Tumor microenvironmental remodeling	A regulator of migration through targeting the tight junction protein ZO-1	(25)
Cancer-associated fibroblast derived exosomes	nutrients	amino acids, lipids, and TCA-cycle intermediates	Tumor microenvironmental remodeling	Inhibit mitochondrial oxidative phosphorylation	(28)
Bladder cancer cells	Casein kinase II α and annexin A2	Protein	EMT	Promote EMT	(34)
Lung adenocarcinoma cell	miR-499a-5p	miRNA	EMT	The proliferation, EMT and migration of lung adenocarcinoma cells are promoted through the M-TOR signaling pathway	(38)
Tumor stem cell	miR-19b-3p	miRNA	EMT	Tumor stem cells cause EMT by expressing the gene PTEN	(39)
Chronic subdurative hematoma	miR-144-5p	miRNA	Angiogenesis	Promotes highly permeable angiogenesis and inhibits hematoma absorption	(40)
Gastric cancer cells	miR-155	miRNA	Angiogenesis	Box-o3 targeting endothelial cells can promote angiogenesis in gastric cancer tissues	(41)
Bone marrow mesenchymal stem cells	unknown	unknown	Angiogenesis	Promote the survival of flaps and reduce the occurrence of necrosis	(42)
Nasopharyngeal carcinoma cells	miR-17-5p	miRNA	Angiogenesis	Targeting BAMBI by regulating the AKT/VEGF-A signaling pathway promotes angiogenesis and proliferation and migration of nasopharyngeal carcinoma cells	(43)
Ovarian cancer cells	miR-205	miRNA	Angiogenesis	It promotes the metastasis of tumor cells by causing vascularization	(44)
Pancreatic cancer cell	miR-27a	miRNA	Angiogenesis	The angiogenesis of human microvascular endothelial cells was promoted by BTG2	(45)
Breast cancer cell	S100	Protein	Angiogenesis	Activation of Src kinase signaling pathway promotes pulmonary vascular leakage	(46)
M2-type macrophages	miR-21-5p&miR-155-5p	miRNA	Invasion and migration	It can combine with BRG1 encoding sequence, down-regulate the expression of BRG1, and promote the invasion and migration of colorectal cancer	(47)
Colorectal cancer cell	Calcium-dependent activator protein for secretion 1 (CAPS1)	Protein	Migration	Normal epithelial FHC cell migration was promoted	(48)
HCC827 cells	MET	mRNA	Invasion and migration	Mediates the invasion and migration of non-small cell lung cancer	(49)
Liver cancer cells	CLEC3B	DNA	Metastasis and angiogenesis	HCC metastasis, EMT, and angiogenesis are mediated through AMPK and VEGF signaling pathways	(50)
Glioblastoma	L1CAM	Protein	Invasion	Stimulate movement, invasion, and proliferation of glioblastoma	(51)
Cancer-associated fibroblasts	miR-382-5p	miRNA	Invasion and migration	Promote the invasion and migration of oral squamous cell carcinoma	(52)
Lymphatic endothelial cells	miR-503-3p、miR-4269&miR-30e-3p	miRNA	Metastasis	Regulate the tumor microenvironment and tumor communication between key molecules and promote the metastasis of breast cancer	(53)
Breast cancer cells	CD47	Protein	Immune regulation and tumor metastasis	It may mediate the immune escape of macrophages and T cells, create a tumor metastasis microenvironment for the metastasis, migration and invasion of tumors, and enable tumor cells to escape the recognition, killing and phagocytosis by T cells and NK nuclear macrophages	(54)
Liver cancer cells	CD81	Protein	Immune escape and tumor metastasis	Promotes liver cancer cells metastasis in HCC caused by viral hepatitis C	(55)

## Tumor Cell-Derived Exosomes and Angiogenesis

Abundant vascular tissue is often found in tumor tissue. Inhibiting tumor angiogenesis is an important part of the cancer treatment process. Tumor cells can secrete a large amount of angiogenic growth factor, leading to the formation of irregular vascular networks in tumor tissues (56). Bone marrow mesenchymal stem cell-derived exosomes can stimulate bone regeneration by regulating ossification and angiogenesis (57, 58). Exosomes from chronic subdural hematomas promote highly permeable angiogenesis and inhibit hematoma absorption through miR144-5P (40). In the tumor microenvironment, tumor cell-derived exosomes serve as a vehicle for intercellular communication. For instance, Zhou Z et al. found that exosome-loaded miR-155 targeting Box O3 of endothelial cells can promote angiogenesis in gastric cancer (41). In addition to tumor cells, exosomes derived from adipose stem cells can also promote angiogenesis after flap transplantation, thus improving the survival rate of flaps (59). Additionally, exosomes from bone marrow mesenchymal stem cells can improve the expression of VEGF and CD34 by local injection, thus promoting the survival of flaps and reducing the occurrence of necrosis (42).

Exosome-derived miR-17-5p can promote angiogenesis in nasopharyngeal carcinoma by targeting BAMBI, and can promote proliferation and migration of nasopharyngeal carcinoma cells by regulating the AKT/VEGF-A signaling pathway (43). He L et al. found increased expression levels of miR-205 in ovarian cancer tissues, adjacent tissues, and serum of patients, and confirmed that miR-205 in exosomes derived from ovarian cancer cells could promote the metastasis of tumor cells by inducing vascularization (44). Pancreatic cancer cell-derived exosomes containing miR-27a promotes the vascularization of human microvascular endothelial cells through BTG2, thus promoting the occurrence and development of pancreatic cancer (45). By upregulating a subgroup of S100 proteins and activating the Src kinase signaling pathway, human breast cancer cell-derived exosomes are able to promote pulmonary vascular leakage (46).

In summary, exosomes secreted by different tumor cells play a role in promoting angiogenesis. When tumor cells reach new metastasis sites, exosomes derived from tumor cells can promote the formation of new blood vessels and promote the growth of tumor cells at metastasis sites (**Figure 1**).

## Relationship Between Tumor Cell-Derived Exosomes and Cancer Cell Migration, Invasion and Metastasis

Cell migration refers to the ability of a cell to move when it receives a migration signal, or when it senses a change in the concentration of some substances, such as enzymes or RNA (60, 61). Cancer cell migration, as it is known, is an important step in tumor metastasis.

Lan J et al. found that exosomes secreted by M2-type neutrophils regulate the invasion and migration behavior of colorectal cancer cells, which are rich in overexpressed miR-21-5p and miR-155-5p that bind to the coding sequence of *BRG1* and lead to a decrease in *BRG1* expression, thus playing an important role in the development of colorectal cancer (47).Tumor cell-derived exosomes containing CEMIP proteins promote cancer cell colonization in brain metastases. By upregulating the pro-inflammatory cytokines encoded by Tnf, Ptgs2 and Ccl/Cxcl, the uptake of CEMIP-positive exosomes by brain endothelial cells and microglia can induce differentiation and inflammation of perivascular endothelial cells, thereby promoting vascular remodeling and tumor metastasis. Moreover, elevated levels of CEMIP in tumor tissues and exosomes from patients with brain metastasis can predict the progression of brain metastasis and patient survival (62). Wu B et al. found that exosomes isolated from colorectal cancer cells with overexpression of calcium-dependent activator protein secretion factor 1 (CAPS1) promoted the migration of normal epithelial FHC cells, and the expression of bone morphogenesis protein 4 decreased in exosomes, which could be helpful for the treatment of patients with metastatic colorectal cancer (48). Yu Y et al. found that HCC827 cells resistant to Icotinib could produce exosomes containing the MET oncogene and could mediate the invasion and migration of non-small cell lung cancer. Downregulation of MET in exosomes could significantly reduce the invasion and migration of HCC827 cells (49). In addition, another study found that downregulation of exosome-derived CLEC3B can promote liver cancer metastasis, EMT, and angiogenesis through the AMPK and VEGF signaling pathways, and suggested that CLEC3B in exosomes may be a new prognostic factor and a potential therapeutic target for liver cancer (50).

Exosomal L1CAM (immunoglobulin superfamily protein), can stimulate glioblastoma movement, invasion, and proliferation, resulting in a poorer prognosis for patients with glioma (51). Exosomal miR-382-5p derived from CAFs is overexpressed compared with adjacent normal tissues, and can promote the invasion and migration of oral squamous cell carcinoma (52). Studies have shown that large amounts of integrins and matrix metalloproteinases (MMPs) enhance basement membrane degradation (63). There is evidence that tumor cell-derived exosomes can migrate to new metastatic niches (also called pre-metastatic niches) by targeting specific receptor cells. Liposomes and membrane proteins are involved in organic metastasis of tumor-derived exosomes. For example, tumor cell-derived exosomes can carry α6β4 and α6β1 integrins targeting the lungs, while αvβ5 integrin targets the liver. In addition, after reaching new tissues and organs, exosomes play a role in the establishment and development of the pre-metastatic ecological niche (64). Based on miRNA expression profiling and functional analysis of lymphatic endothelial cells (LECs), Kim et al. identified miR-503-3p, miR-4269, and miR-30e-3p as downstream targets of ELK3 in LECs. The expression of ELK3 in LECs promotes breast cancer progression and metastasis, suggesting that ELK3 is a key factor in regulating the tumor microenvironment and tumor-to-tumor communication and promoting cancer metastasis (53).

In conclusion, the above studies suggest that tumor cell-derived exosomes regulate the metastasis and migration of tumor cells through different types of proteins and miRNAs (**Table 1**).

## Relationship Between Tumor Cell-Derived Exosomes and Immune Regulation

The activity of exosomes affects five major functions of immune regulatory mechanisms: immune activation, antigen expression, immune surveillance, immunosuppression, and communication between immune cells. In addition to immune cells, tumor cells can also secrete immunoreactive exosomes, thus influencing pathophysiological processes (65–67). Furthermore, there is increasing evidence that exosomes may contribute to cancer development by regulating different immune cells (68, 69) (**Figure 1**).

Seo N et al. found that dendritic cells (DCs) could promote CTL generation, while Treg-cell-derived exosomes could inhibit CTL generation, and that exosomes from immune cells and tumor cells could regulate cancer progression (70). Tumor cell-derived exosomes enhance the secretion of prostaglandin E2, TGF-β, and IL-6 in myeloid cells, resulting in a strong immunosuppressive environment in tumor lesions (71, 72). CD47, a glycoprotein on the surface of the cell membrane, is a member of the immunoglobulin (Ig) family and one of the cell membrane receptors that can be exploited in immunotherapy. CD47, as an inhibitory receptor on the surface of tumor cells, can interact with the signaling protein SIPR-α on the surface of the cell membrane, which may mediate immune escape from macrophages and T cells. In addition, CD47 is highly expressed in tumor cell-derived exosomes, can create a tumor microenvironment, lay the foundation for tumor metastasis, migration and invasion, and enable tumor cells to escape T cells and NK nuclear macrophages, thus promoting the occurrence and development of tumors (54). CD81, an immunomodulator, can promote tumor progression and promote the metastasis of HCC caused by hepatitis C virus (HCV). Ashraf Malik M et al. found that the CD81^+^ exosomes carried HCV particles and established an environment for persistent HCV infection to promote the progression of liver cancer through immune escape (55).

In addition to exosomes regulating tumor immune escape, proteins and nucleic acids contained in exosomes can also be used as substances detected by immune checkpoint blockade technology. Due to previous technical limitations, exosome extraction has often been difficult. However, with the development of microfluidic technology, RNA sequencing technologies, and the rapid development of proteomics, the use of exosomes as a diagnostic and prognostic biomarker for immune checkpoint blockade technology has become possible (73).

## Tumor Cell-Derived Exosomes and Tumor Therapy

As a treatment option, exosomes are characterized by the lack of toxic side effects and rejection, and can therefore be used as a vehicle for drug delivery in cancer therapy. Exosomal delivery of adriamycin and paclitaxel that has been used in targeted cancer therapy with less toxic side effects and immunogenicity (74, 75). In addition, exosome-derived miRNA may be significant in the metabolism, diagnosis, and treatment of cancer. The metabolism of tumor cells from many different tissues requires the involvement of mitochondria, and exosomes can trigger metabolic reprogramming and inhibit tumor growth by restoring tumor cell respiration. Therefore, exosome-derived miRNAs, by mediating the metabolism of tumor cells, may have some values in the prognosis and treatment of cancer (76).

Tumor stem cells are believed to be the seed cells of the primary cancer and the source of drug resistance to radiotherapy and chemotherapy. The accurate delivery of drugs to tumor stem cells is the current focus and challenge facing cancer therapy. Nanotechnology combined with exosomal drug delivery has the potential to address this challenge and potentially improve the efficacy and specificity of targeted cancer stem cell therapy (77). The exosome expression profile of patients with multiple myeloma is different from that of healthy patients, and may have potential therapeutic effects in patients with multiple myeloma. In addition, tumor cell-derived exosomes and their byproducts can be edited and modified to produce anti-tumor vaccines (78). Nie W et al. synthesized exosome nano-bio-conjugates through biosynthesis and, after systemic administration, specifically identified aCD47 and CD47 on the surface of tumor cells, demonstrating that nano-bio-conjugates could actively target tumor cells (79).

Exosomes may also provide solutions to long-standing drug delivery challenges. While aspirin was found to have anti-tumor effects, the challenge of delivering it to the tumor has limited its application (80). In order to solve the problem of poor water-solubility of aspirin, low exosome inclusion rate, and to further develop new anticancer drugs from aspirin, TranPHL et al. developed and established a nanocrystalline exosome transport and delivery platform. In vitro and *in vivo* studies for the treatment of breast cancer and colorectal cancer have shown that these exosomes enhance cell uptake through clathrin-dependent and independent endocytic pathways, significantly improve the cytotoxicity of aspirin to breast cancer and colorectal cancer cells, and enhance tumor cell apoptosis and autophagy. In addition, aspirin encapsulated by nanocrystalline exosomes has an unprecedented ability to remove tumor stem cells (81) (**Figure 1** and **Table 2**).

**Table 2:** Exosomes and tumor therapy.

Exosomes type	Function in tumor therapy	Reference
The carrier	Exosomes serve as carriers for doxorubicin and paclitaxel, which can be used in targeted cancer therapy.	(74, 75)
Nano-bioconjugates	Nano engineering technology combined with exosomes for drug delivery, targeting tumor stem cells. aCD47 and CD47 on the surface of tumor cells are specifically recognized, and nano-bio-conjugate can actively target tumor cells.	(77, 79)
Protein, miRNA, etc.	It is possible to make anti-tumor vaccines, by modifying the products of tumor-derived exosomes.	(78)
Aspirin-loaded nano-exosomes	Cell uptake was enhanced by clathrin-dependent and independent endocytic method, and the cytotoxicity of aspirin on breast cancer and colorectal cancer cells was significantly improved, while tumor cell apoptosis and autophagy were enhanced. Aspirin-loaded nano-exosomes has an unprecedented ability to remove tumor stem cells.	(81)

## Conclusion and Perspectives

Exosomes are extracellular vesicles that contain nucleic acids, proteins, lipids and other substances. Compared with tumor markers that exist in tissues and body fluids, exosomes are not only highly stable but are also rich in content, which lays a solid foundation for the future clinical application of exosomes (26). The extraction, isolation, and identification of tumor cell-derived exosomes may contribute to the following aspects. Firstly, exosomes may be used to elucidate the underlying mechanisms of tumor progression and provide a potential therapeutic target for cancer patients. Secondly, exosomes can be used to treat cancers by encapsulating some antitumor drugs. Thirdly, tumor immunotherapy has attracted more and more attention, and the immunoregulatory properties of exosomes include regulation of antigen presentation, immune supervision, and immune activation. An in-depth study on the molecular mechanisms of interaction between exosomes and immune cells may reveal a new approach for tumor immunotherapy (82).

Exosomes are versatile and serve a critical function in intercellular communication. In this article, we elaborated on exosome-mediated tumor metastasis, tumor angiogenesis, EMT, exosome immunomodulatory functions, and the role of combining nanoengineering technologies to fight cancer. Future studies may focus on the potential heterogeneity of tumor cell-derived exosomes, which will help to understand the clonal expansion of tumor cells and the characteristics of clonal expansion after cancer treatment (6). By using sequencing technologies, proteomics, and the detection of RNA and DNA in serum exosomes will hopefully be used for early diagnosis and prognostic evaluation of cancer.

As an intermediate of intercellular communication, exosomes play a vital role in the pathogenesis, diagnosis, and treatment of tumors. However, there are still a number of problems that need to be addressed before its clinical application. First, how do we identify exosomes? To address this problem, a relevant study has reported that Alix, CD9, TSG101, and CD63 are protein markers of exosomes (83). However, not all tissues and cells express these so-called exosome markers, as a result of the specificity of tissues and cells. Therefore, further investigation into how exosomes are identified is an important objective of future research. In addition, we need to pay attention to the differences between exosomes and microvesicles. Microvesicles are larger in diameter than exosomes. The origin of exosomes is the endosome, while the origin of microvesicles is the plasma membrane. In addition, the two are also morphologically different (3). This reminds us that we must pay attention to distinguish and identify exosomes in future studies. Second, how do we extract exosomes? Pin Li et al. summarized how to use the physical, chemical and biochemical properties of exosomes to extract exosomes, including ultrafiltration-based isolation techniques, size-based isolation techniques, immunoaffinity capture-based techniques, among others (84). In his summary, the author suggests that ultrafiltration is a method of exosome extraction that has great potential, especially in the treatment and analysis of exosomes isolated from human blood and plasma (84).

The ultimate service of basic medicine is the patient. According to the clinical needs, how to extract exosomes quickly and effectively and apply them to the diagnosis and treatment of cancer patients should be a priority to the field and a major objective of future research. In addition, a number of unknown molecular mechanisms, such as how exosomes mediate cancer progression and tumor suppression, and immature artificial exosome synthesis techniques have brought new challenges to exosomes in the diagnosis, treatment, and prognostic guidance of cancer.

## Author Contributions

YL wrote the essay. KS and YC made tables. YL, XW and ZC drew diagram. KC and YT revised the introduction and the first half of the article. JL helped to revise the conclusion. XC and JZ helped to revise the framework of the article. All authors contributed to the article and approved the submitted version.

## Conflict of Interest

The authors declare that the research was conducted in the absence of any commercial or financial relationships that could be construed as a potential conflict of interest.
